# Supplementary material for: Neuropathological Applications of Microscopy with Ultraviolet Surface Excitation (MUSE): A Concordance Study of Human Primary and Metastatic Brain Tumors
Source: Brain Sci. 2024 Jan 22;14(1):108. doi: 10.3390/brainsci14010108 (PMC10813539; doi:10.3390/brainsci14010108)
Supplement: Supplementary file 1 [file brainsci-14-00108-s001.zip › brainsci-2767605-supplementary.pdf]

Supplementary Files

Table S1. Normalized RNA read counts across 7 control genes.

		Sample 39N				Sample 53			
		MUSE		Fresh frozen		MUSE		Fresh frozen	
**Gene**	**Amplicon**	Read Count	RPM	Read Count	RPM	Read Count	RPM	Read Count	RPM
ALK	ALK.3p.E22E24	33	113.1	20	103.3	17	49.2	55	140.2
TBP	TBP.ENCTRL.E3E5	62984	215837.6	13820	71349.7	107946	312475.8	133915	341469.2
MRPL13	MRPL13.ENCTRL.E1.E3	65388	224075.8	4556	23521.6	121735	352391.3	97503	248622.4
MYC	MYC.ENCTRL.E2E4	17708	26121.4	21231	37164.5	56962	59510.2	61665	75666.1
HMBS	HMBS.ENCTRL.E8E10	31313	46190.4	6194	10842.5	33184	34668.5	32703	40128.3
ITGB7	ITGB7.ENCTRL.E14E16	14499	21387.7	69743	122083.9	11129	11626.9	20522	25181.5
LRP1	LRP1.ENCTRL.E57E59	73023	250239.9	20936	108088.0	60399	174839.5	91739	233924.8

Note: This table compares RNA read data from samples 39N and 53 (fresh frozen tissue), and 39N-MUSE and 53-MUSE (MUSE treated tissue). RNA sequencing was performed using the Oncomine Comprehensive Assay version 3 (OCAv3). Shown in the table are read counts for each amplicon analyzed and normalized read counts, expressed as Reads Per Million (RPM).

A

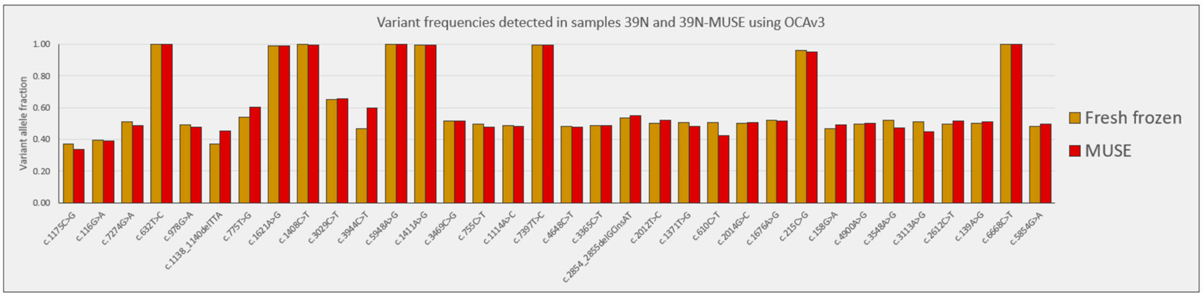

B

				Sample 39N			
				Fresh frozen		MUSE	
Transcript ID	Gene	Variant (DNA)	Protein	VAF	Coverage	VAF	Coverage
NM_001033082.2	*MYCL*	c.1175C>G	p.Thr392Ser	0.37	3040	0.34	3625
NM_000179.2	*MSH6*	c.116G>A	p.Gly39Glu	0.40	2051	0.39	2143
NM_001184.3	*ATR*	c.7274G>A	p.Arg2425Gln	0.51	6993	0.49	5491
NM_001184.3	*ATR*	c.632T>C	p.Met211Thr	1.00	7266	1.00	6590
NM_181523.2	*PIK3R1*	c.978G>A	p.Met326Ile	0.49	4007	0.48	3317
NM_181523.2	*PIK3R1*	c.1138_1140delTTA	p.Leu380del	0.37	3480	0.45	3509
NM_001760.4	*CCND3*	c.775T>G	p.Ser259Ala	0.54	6003	0.60	4394
NM_000535.6	*PMS2*	c.1621A>G	p.Lys541Glu	0.99	3744	0.99	3832
NM_000535.6	*PMS2*	c.1408C>T	p.Pro470Ser	1.00	13382	1.00	12500
NM_001127500.2	*MET*	c.3029C>T	p.Thr1010Ile	0.65	2609	0.65	2438
NM_000264.3	*PTCH1*	c.3944C>T	p.Pro1315Leu	0.47	2009	0.60	1745
NM_000051.3	*ATM*	c.5948A>G	p.Asn1983Ser	1.00	4560	1.00	3059
NM_001274.5	*CHEK1*	c.1411A>G	p.Ile471Val	1.00	3667	1.00	2773
NM_006231.3	*POLE*	c.3469C>G	p.Pro1157Ala	0.52	3086	0.52	3041
NM_006231.3	*POLE*	c.755C>T	p.Ala252Val	0.50	3953	0.48	3785
NM_000059.3	*BRCA2*	c.1114A>C	p.Asn372His	0.49	4459	0.48	3080
NM_000059.3	*BRCA2*	c.7397T>C	p.Val2466Ala	1.00	3521	1.00	2743
NM_032444.3	*SLX4*	c.4648C>T	p.Arg1550Trp	0.48	5738	0.48	4802
NM_032444.3	*SLX4*	c.3365C>T	p.Pro1122Leu	0.49	7752	0.49	6140
NM_032444.3	*SLX4*	c.2854_2855delGCinsAT	p.Ala952Met	0.54	2461	0.55	2428
NM_032444.3	*SLX4*	c.2012T>C	p.Leu671Ser	0.50	3658	0.52	3515
NM_032444.3	*SLX4*	c.1371T>G	p.Asn457Lys	0.51	6073	0.48	3305
NM_032444.3	*SLX4*	c.610C>T	p.Arg204Cys	0.51	4008	0.42	2502
NM_024675.3	*PALB2*	c.2014G>C	p.Glu672Gln	0.50	2661	0.51	2745
NM_024675.3	*PALB2*	c.1676A>G	p.Gln559Arg	0.52	3362	0.52	2432
NM_000546.5	*TP53*	c.215C>G	p.Pro72Arg	0.96	6646	0.95	4589
NM_133629.2	*RAD51D*	c.158G>A	p.Arg53Gln	0.47	5214	0.49	5229
NM_007300.3	*BRCA1*	c.4900A>G	p.Ser1634Gly	0.50	5817	0.50	5216
NM_007300.3	*BRCA1*	c.3548A>G	p.Lys1183Arg	0.52	6988	0.47	4640
NM_007300.3	*BRCA1*	c.3113A>G	p.Glu1038Gly	0.51	5569	0.45	5109
NM_007300.3	*BRCA1*	c.2612C>T	p.Pro871Leu	0.50	4937	0.52	633
NM_017763.5	*RNF43*	c.139A>G	p.Ile47Val	0.50	1570	0.51	969
NM_000435.2	*NOTCH3*	c.6668C>T	p.Ala2223Val	1.00	6432	1.00	6506
NM_000435.2	*NOTCH3*	c.5854G>A	p.Val1952Met	0.48	2392	0.50	2845

Figure S1. Comparison of variants identified in samples 39N and 39N-MUSE using amplicon-based OCAv3. Note: This file compares the variants identified in paired fresh frozen (39N) and MUSE-treated (39N-MUSE) samples. Variants were identified using the Oncomine Comprehensive Assay version 3 (OCAv3). **A**, Histogram showing the variant allele frequencies (VAF) for each variant in both tissues. **B**, Table listing each variant. Variants are described using Human Genome Variation Society (HGVS) nomenclature, including the RefSeq transcript identifier. Coverage represents total read coverage.
